# Leptin Signaling in Kiss1 Neurons Arises after Pubertal Development

**DOI:** 10.1371/journal.pone.0058698

**Published:** 2013-03-07

**Authors:** Roberta M. Cravo, Renata Frazao, Mario Perello, Sherri Osborne-Lawrence, Kevin W. Williams, Jeffery M. Zigman, Claudia Vianna, Carol F. Elias

**Affiliations:** 1 Department of Internal Medicine, Division of Hypothalamic Research, The University of Texas Southwestern Medical Center, Dallas, Texas, United States of America; 2 Department of Anatomy, Institute of Biomedical Sciences, University of São Paulo, São Paulo, Brazil; 3 Laboratory of Neurophysiology, Multidisciplinary Institute of Cell Biology (CONICET/ CICPBA), La Plata, Argentina; 4 Department of Molecular and Integrative Physiology, University of Michigan, Ann Arbor, Michigan, United States of America; University of Cordoba, Spain

## Abstract

The adipocyte-derived hormone leptin is required for normal pubertal maturation in mice and humans and, therefore, leptin has been recognized as a crucial metabolic cue linking energy stores and the onset of puberty. Several lines of evidence have suggested that leptin acts via kisspeptin expressing neurons of the arcuate nucleus to exert its effects. Using conditional knockout mice, we have previously demonstrated that deletion of leptin receptors (LepR) from kisspeptin cells cause no puberty or fertility deficits. However, developmental adaptations and system redundancies may have obscured the physiologic relevance of direct leptin signaling in kisspeptin neurons. To overcome these putative effects, we re-expressed endogenous LepR selectively in kisspeptin cells of mice otherwise null for LepR, using the Cre-loxP system. Kiss1-Cre LepR null mice showed no pubertal development and no improvement of the metabolic phenotype, remaining obese, diabetic and infertile. These mice displayed decreased numbers of neurons expressing *Kiss1* gene, similar to prepubertal control mice, and an unexpected lack of re-expression of functional LepR. To further assess the temporal coexpression of *Kiss1* and *Lepr* genes, we generated mice with the human renilla green fluorescent protein (hrGFP) driven by *Kiss1* regulatory elements and crossed them with mice that express Cre recombinase from the *Lepr* locus and the R26-tdTomato reporter gene. No coexpression of Kiss1 and LepR was observed in prepubertal mice. Our findings unequivocally demonstrate that kisspeptin neurons are not the direct target of leptin in the onset of puberty. Leptin signaling in kisspeptin neurons arises only after completion of sexual maturation.

## Introduction

Many aspects of reproductive physiology are energetically demanding (e.g., pregnancy and lactation); therefore, the nutritional state is a critical factor for pubertal development and attainment of reproductive capacity [1]–[3]. Previous studies in rats have indicated that the time of pubertal onset is correlated with body size, not chronological age [4], and epidemiological data in humans suggested that a critical amount of body fat is required for proper sexual maturation [5]. Subsequently, a series of studies in different species has demonstrated that conditions of extreme leanness delay the initiation and progression of pubertal maturation [2], [6].

Following the discovery of leptin primarily as an adipocyte-derived hormone and its requirement for pubertal development in mice and humans, leptin was recognized as a potential link between energy stores and the onset of puberty [7]–[9]. However, the mechanism by which leptin exerts its effect is still a matter of debate. Leptin receptors (LepR) are highly expressed in arcuate nucleus (Arc) neurons, where they partially colocalize with kisspeptin, one of the most potent regulators of the reproductive axis [10]–[13]. Loss-of-function mutations in kisspeptin (*Kiss1/KISS1*) or kisspeptin receptor (*Gpr54/GPR54*) genes cause infertility due to lack of pubertal maturation and hypogonadotropic hypogonadism in mice and humans [14]–[18]. *Kiss1* and *Gpr54* gene expression increases across pubertal development, and the administration of kisspeptin to juvenile rodents precipitates vaginal opening (an external sign of puberty onset) and induces LH secretion and ovulation [19], [20]. Leptin administration to leptin-deficient infertile *ob/ob* mice induces sexual maturation and increases hypothalamic *Kiss1* gene expression [10]. Together, these findings pointed to a prime role for kisspeptin neurons in mediating the permissive action of leptin in the onset of puberty [2], [10], [21]. To test this model, we previously used a Cre conditional LepR knockout mouse model to selectively delete LepR from kisspeptin expressing neurons [22]. Male and female Kiss1-Cre LepR*^lox/lox^* mice showed normal sexual maturation, fertility and fecundity, suggesting that leptin action in Kiss1 neurons is not necessary for puberty and reproduction. However, developmental changes and/or neuronal circuitry redundancies may have obscured the physiological role of leptin signaling on kisspeptin neurons. To clarify this, we re-expressed endogenous LepR selectively in Kiss1 neurons and determined whether leptin activity only in Kiss1 cells is sufficient to allow pubertal development and to rescue the fertility of the LepR null mice.

## Materials and Methods

### Animals

Male and female Kiss1-Cre, LepR null, LepR-IRES-Cre, R26 GFP or R26 tdTomato (B6.Cg-Tg(ACTB-Bgeo/GFP)21Lbe/J; B6.Cg-Gt(ROSA)26Sortm14(CAG-tdTomato)Hze/J, Jax® mice), Kiss1-hrGFP and C57BL/6 mice were housed in the University of Texas Southwestern Medical Center Animal Resource Center, in a light- (12 h on/12 h off) and temperature- (21–23°C) controlled environment with free access to water and food. Groups of females were assessed daily for vaginal opening or estrous cycles and were bilaterally ovariectomized for 7–10 days before perfusion. All experiments were carried out in accordance with the guidelines established by the National Institute of Health Guide for the Care and Use of Laboratory Animals. All experiments and procedure in this study were approved by the University of Texas Institutional Animal Care and Use Committee (IACUC, AP#2008-0150).

*Metabolic Phenotyping:* Mice were maintained on a standard chow diet (Harlan Teklad Global Diet) and were followed from 13 to 35 weeks of age. Body weight was determined weekly. Body composition (total lean and fat mass) was assessed at 20, 28 and 35 weeks of age by magnetic resonance imaging using an EchoMRI-100 quantitative NMR machine. Body composition is expressed as a percentage of body weight. Basal glucose levels were determined in fed and overnight fasted mice.

### DNA recombination – PCR strategy

Oligonucleotide primers were designed to confirm the DNA recombination sites: LepReacRev: 5′-TAGGGCCAAACCCACATTTA-3′; LepReacFor: 5′-CAGTCTGGACCGAAGGTGTT-3′; pDisR: 5′-CCCAAGGCCATACAAGTGTT-3′. Selected tissues were submitted to 36 PCR cycles using the following temperatures: 94°C (denaturation, 30′); 58°C (annealing, 30′) and 72 °C (extension, 60′). The full length and Cre-mediated deletion of the *loxP*-flanked transcription-blocking cassette were distinguished according to size/base pairs (540bp  =  full length, 300bp  =  DNA recombination).

### Whole-Cell recording

Whole-cell patch-clamp recordings were performed in Kiss1 neurons of the preoptic area (AVPV/PeN, n = 18) and the Arc (n = 30) of adult (P60-70) males and females on diestrus. Neurons were maintained in hypothalamic slice preparations, and data analyses were performed as previously described [23], [24]. Epifluorescence and differential interference contrast imaging (DIC) was used to target Kiss1 neurons (Nikon Eclipse FN1 and a QuantEM:512SC electron-multiplying charge-coupled device camera). Electrophysiological signals were recorded using an Axopatch 700B amplifier (Molecular Devices), low-pass filtered at 2–5 kHz, digitized at 88 kHz (Neuro-corder; Cygnus Technology), and analyzed offline on a PC with pCLAMP programs (Molecular Devices). Recording electrodes had resistances of 2.5–5 MΩ when filled with the K-gluconate internal solution. Membrane potential values were compensated to account for junction potential (-8 mV). Solutions containing leptin were perfused for 5 min.

### Generation and Validation of Kiss1-hrGFP mice

The Kiss1-hrGFP mouse model (or KG) was generated as previously described [25] using the BAC RP24-299B2, except that a DNA fragment containing the coding sequence of hrGFP was used instead of the Cre sequence. The hrGFP-modified *Kiss1* BAC was submitted to the UTSW Medical Center Transgenic Core Facility for microinjection into the pronuclei of fertilized one-cell stage embryos of C57BL/6 mice. Oligonucleotide primers used to confirm the genotype of mice harboring the Kiss1-hrGFP transgenes were as follows: M358: 5′-GCTCTGGTGAAGTACGAACTCTGA-3′, M243: 5′-AGGTGCGGTTGCCGTACTGGA-3′, M176: 5′-GGTCAGCCTAATTAGCTCTGTCAT-3′ and M117: 5′-GATCTCCAGCTCCTCCTCTGTCT-3′. Validation of our mouse model was performed in ovariectomized and ovariectomized estrogen-primed females (n = 3/group) by colocalization of Kiss1 mRNA (in situ hybridization) in neurons immunoreactive to hrGFP (1:2000, Stratagene-Agilent).

### Histology

Mice were deeply anesthetized with chloral hydrate (7%, ip) and perfused with 10% buffered formalin. Brains were sectioned (25-µm sections, 5 series) in the frontal plane. GFP immunoreactivity (GFP-ir) was assessed in series of brain sections from both male and female Kiss1-Cre reporter mice. Sections were incubated in primary anti-GFP (1:5,000, Aves Labs) overnight and in AlexaFluor 488-conjugated goat anti-chicken secondary antisera (1∶250, Invitrogen) for 1 h. Hypothalamic sections from mice treated with leptin or saline were incubated in antisera against pSTAT3 (1∶2,000, Cell Signaling), Fos (1∶10,000, Millipore) or pSTAT5 (1∶1,000, Cell Signaling) at 4°C for 48–72 h. Sections were incubated in biotin-conjugated donkey anti-rabbit secondary antisera (1∶1,000, Jackson Laboratories) and avidin-biotin complex (1∶500, Vector Labs) and subjected to immunoperoxidase reaction using diaminobenzidine as chromogen. Dual label in situ hybridization /immunohistochemistry was performed to determine coexpression of GFP-ir and LepR mRNA. The procedure was a modification of that previously reported [25], [26]. The LepR riboprobe was generated by *in vitro* transcription with ^33^P-UTP. Hybridization signals were detected following standard autoradiographic protocols [26]. Brain sections were analyzed using a Zeiss Axioplan microscope. Quantification of single- or dual-labeled neurons and percentage of colocalization were determined in the AVPV, PeN and in 1 or 2 rostro-to-caudal levels of the Arc. Cells were counted in one side of a defined level of each nucleus. For the in situ hybridization, we considered cells dual labeled if the density of silver grains (hybridization signal) overlying a cell was at least 3 × that observed in the background.

### Statistical Analysis

Data are expressed as the mean ± SEM. Comparisons between two groups were carried out using the unpaired two-tailed Student’s *t* test. One-way ANOVA followed by the pairwise Tukey test were used to compare three or more groups simultaneously. Statistical analysis was performed using GraphPad Prism software, and an α value (*P*) of 0.05 was considered significant in all analyses.

## Results and Discussion

### Generation and validation of Kiss1-Cre LepR null mice

**Figure 1:**
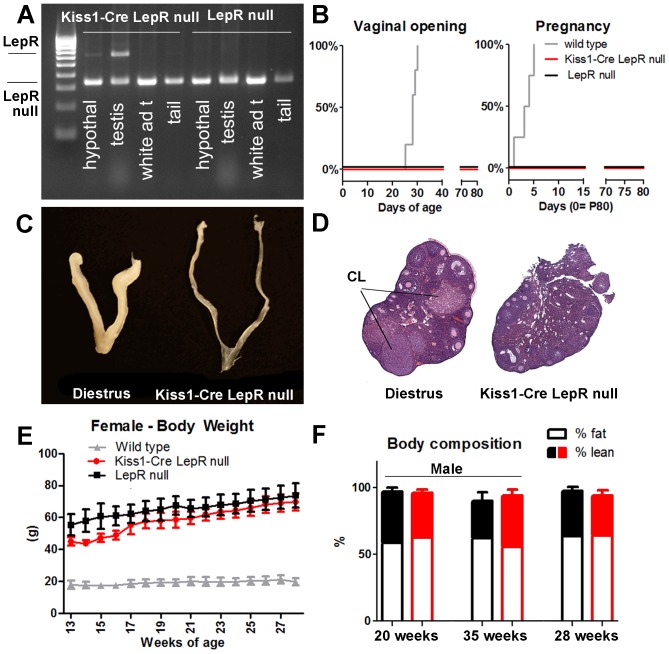
Re-expression of LepR selectively in Kiss1 neurons causes no amelioration of the reproductive or metabolic phenotype of LepR null mice. A. Agarose gel showing Cre-induced DNA recombination (higher band) of LepR*^Lox^* ^TB^ in the hypothalamus and testis (but not in the white adipose tissue and tail) of Kiss1-Cre LepR null mice. B. Survival graphs showing the progression of vaginal opening and pregnancy in wild type, LepR null and Kiss1-Cre LepR null mice; C. Image comparing the size of the uterus of a wild type female on diestrus and adult Kiss1-Cre LepR null mice; D. Image showing sections of the ovary of a female on diestrus and of an adult Kiss1-Cre LepR null female. Note the presence of corpora lutea (CL) only in the ovary of the wild type female mice. E. Graph showing the progression of body weight of wild type, LepR null and Kiss1-Cre LepR null female mice. F. Bar graphs showing body composition (percentage of fat and lean mass) of LepR null (black) and Kiss1-Cre LepR null (red) mice at 3 different ages: 20 weeks, 35 weeks (males) and 28 weeks (females).

To generate mice with endogenous re-expression of LepR selectively in Kiss1 neurons, we crossed Kiss1-Cre (J2-4) mice [25] with the LepR null mice previously described and validated by our group [27]. Briefly, LepR null mice have a *loxP*-flanked transcription-blocking cassette (TB) inserted between exons 16 and 17 of the *Lepr* gene to generate mice lacking the long isoform of LepR. *Lepr^lox^* ^TB*/lox*TB^ mice (LepR null) are obese, diabetic and infertile. Heterozygous (*Lepr^lox^* ^TB/WT^) mice were bred with Kiss1-Cre mice to produce *Kiss1^Cre/-^ Lepr^lox^* ^TB/WT^ male and female mice, which were used as breeders to generate our experimental animals. Genotypes of the resulting progeny were produced at expected Mendelian ratios. Two cohorts of Kiss1-Cre LepR null mice (*Kiss1^Cre/?^ Lepr^lox^* ^TB/*lox*TB^, n = 8–11/group/genotype/sex) were evaluated and compared with LepR null (*Kiss1^-/-^ Lepr^lox^* ^TB/*lox*TB^) and wild type (WT, *Kiss1^-/-^ Lepr^WT^* ^/*WT*^) littermates (n = 4-6/group/genotype/sex). Groups were defined by tail genotyping, and endogenous re-expression of LepR in Kiss1 neurons was assessed at the end of the experiment. The Cre-recombined alleles were initially verified using a PCR strategy in which the presence of full length and Cre-mediated deletion of the *loxP*-flanked transcription-blocking cassette were distinguished (n  =  4 /sex/genotype). Recombination sites were only observed in tissues that express the *Kiss1* gene, including the hypothalamus and gonads (Figure 1A).

### Kiss1-Cre LepR null mice show no improvement in reproductive function

Mice were weaned at 25 days of age and females were monitored for vaginal opening (VO), as a consensus external sign of puberty onset. All WT littermates had VO at the expected age (27–30 days), but no or undeveloped VOs were observed in Kiss1-Cre LepR null and LepR null mice, monitored until P80 (Fig. 1B). Because male odorants may induce sexual maturation in female rodents [28], we further characterized the reproductive phenotype of the female mice in the presence of a C57BL/6 male of proven fertility. Males were introduced in cages containing at least one female of each genotype (Kiss1-Cre LepR null, LepR null and WT). After 5 days, all WT females were impregnated, whereas no Kiss1-Cre LepR null and no LepR null female mice were fertile after 10–12 weeks of mating (Fig. 1B). Kiss1-Cre LepR null and LepR null female mice showed no uterine development and no ovarian corpora lutea, indicating a lack of increase in estrogen levels, LH secretion and ovulation (Fig. 1C–D). Likewise, adult male mice (P80) were housed with 2–3 WT females of proven fertility for 10–12 weeks. All WT males impregnated at least 2 females in 1 week, whereas no Kiss1-Cre LepR null and no LepR null male mice were fertile. No difference in testis weight was observed between Kiss1-Cre LepR null and LepR null littermates (*P*  =  0.92).

### Kiss1-Cre LepR null mice show no improvement in metabolic phenotype

Previous studies have suggested a role for kisspeptin neurons in energy homeostasis [29], we then assessed changes in the metabolic profile of these mice. Male and female Kiss1-Cre LepR null and LepR null mice were similarly obese from 13 to 28 weeks of age (Fig. 1E). No change in fat mass and lean mass was observed (20, 28 and 35 weeks of age, Fig. 1F), and no differences in fed and fasting (overnight) glucose levels were detected between Kiss1-Cre LepR null and LepR null mice (fed: 196.3 ± 43.5 *vs.* 189.8 ± 22.4 mg/dL, *P*  =  0.80 ; fasting: 98.7 ± 11.0 *vs.* 134.3 ± 29.6 mg/dL, *P*  =  0.30).

### Lack of expression of functional LepR in Kiss1 neurons of Kiss1-Cre LepR-null mice

**Figure 2:**
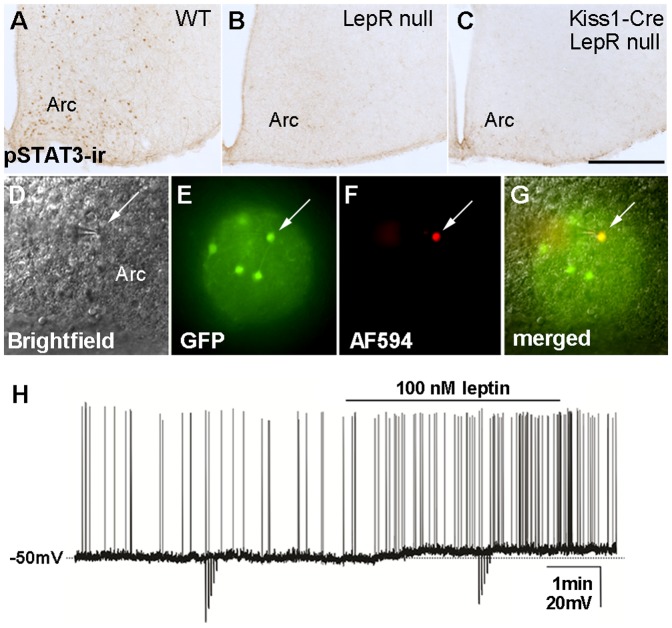
Lack of re-expression of functional LepR in Kiss1-Cre LepR null mice. A-C. Brightfield photomicrographs showing the distribution of leptin-induced phosphorylation of STAT3 immunoreactivity (pSTAT3-ir) in the arcuate nucleus (Arc) of wild type female mice on diestrus (A) and lack of pSTAT3-ir in the Arc of LepR null (B) and of Kiss1-Cre LepR null (C) adult female mice; **D-G.** Identification of Kiss1-Cre/GFP cells for whole-cell patch-clamp recordings. (D) Brightfield illumination showing a targeted neuron; (E) the same neuron under fluorescent (FITC) illumination; (F) complete dialysis of AlexaFluor 594 from the intracellular pipette at the end of the recording; (G) colocalization of AlexaFluor 594 and GFP. **H**. A current-clamp recording demonstrates that leptin (100 nM) depolarizes Kiss1-Cre/GFP neurons. The dashed line indicates the resting membrane potential. Scale bar: A–C  =  400 µm.

To determine the degree of re-expression of functional LepR in Kiss1 neurons, adult mice were 24 h-fasted and received a bolus of intraperitoneal (ip) leptin (2.5 or 5 µg/g) 1 or 2 h before perfusion to assess leptin-induced phosphorylation of signal transducer and activator of transcription-3 (pSTAT3) immunoreactivity [31], [35]. As expected, we observed leptin-induced pSTAT3 in several hypothalamic sites of WT mice and no response in LepR null mice [27]. However, surprisingly, no pSTAT3 was observed in the hypothalamus of Kiss1-Cre LepR-null mice (Fig. 2A–C; 2 different cohorts, n = 4–5/genotype/sex). Because of this unexpected result, additional experimental protocols were used, including ip leptin injections in younger and leaner animals (P15–28 days of age, 5 µg/g, n = 4–5/genotype/sex) or intravenous (jugular vein) administration of leptin (1 g/g, n = 4–5/genotype/sex). Although leptin-injected mice had higher circulating levels of leptin (average > 950 ng/mL *vs.* 200.2 ± 48.9 ng/mL in saline injected mice), none of these manipulations resulted in leptin-induced pSTAT3 in hypothalamic neurons of Kiss1-Cre LepR null mice.

Leptin induces pSTAT3 in virtually all LepR-expressing neurons of the Arc, including those expressing Kiss1 [25], [26]. However, previous studies have suggested that leptin-induced pSTAT3 is not required for reproduction [30]. Thus, we assessed whether leptin engages any alternative previously identified signaling pathway in Kiss1 neurons. For example, leptin recruits the phosphoinositide 3-kinase (PI3K) signaling pathways [31]–[33], and in hypothalamic slices, leptin-induced change in cell activity requires intact PI3K [23], [24]. Moreover, previous studies in guinea pigs have reported that leptin depolarizes high percentage of Kiss1 neurons of the Arc [34]. To evaluate the ability of leptin to change Kiss1 cell activity in WT and Kiss1-Cre LepR null mice, we generated mouse models bearing Kiss1-Cre reporter genes (eGFP or tdTomato, Jax® mice). In contrast to reports in guinea pigs, leptin (100 nM) depolarized only 10% of Arc Kiss1 neurons in the WT mouse (3 out of 30 recorded cells, 5.0 ± 0.9 mV; Fig. 2D–H). As predicted by the lack of LepR expression in Kiss1 neurons of the preoptic area (23), no leptin-induced change in cell activity was observed in Kiss1 neurons of the anteroventral periventricular nucleus or anterior periventricular nucleus of the hypothalamus (AVPV/PeN, n = 18 recorded cells). Because of the low rate of leptin-responsive Kiss1 neurons in the mouse brain, Kiss1-Cre LepR null mice bearing the reporter gene were not used for recordings. Alternatively, we further evaluated the expression of Fos immunoreactivity as an indirect measurement of leptin-induced neuronal activation, and phosphorylation of STAT5 as an additional marker of leptin signaling [33], [35]–[37]. No leptin-induced Fos or pSTAT5 immunoreactivities were observed in the hypothalamus of Kiss1-Cre LepR null mice. These findings suggested that functional LepR has not been re-expressed in Kiss1 neurons of Kiss1-Cre LepR null mice.

### Leptin signaling in Kiss1 neurons arises after completion of sexual maturation

**Figure 3:**
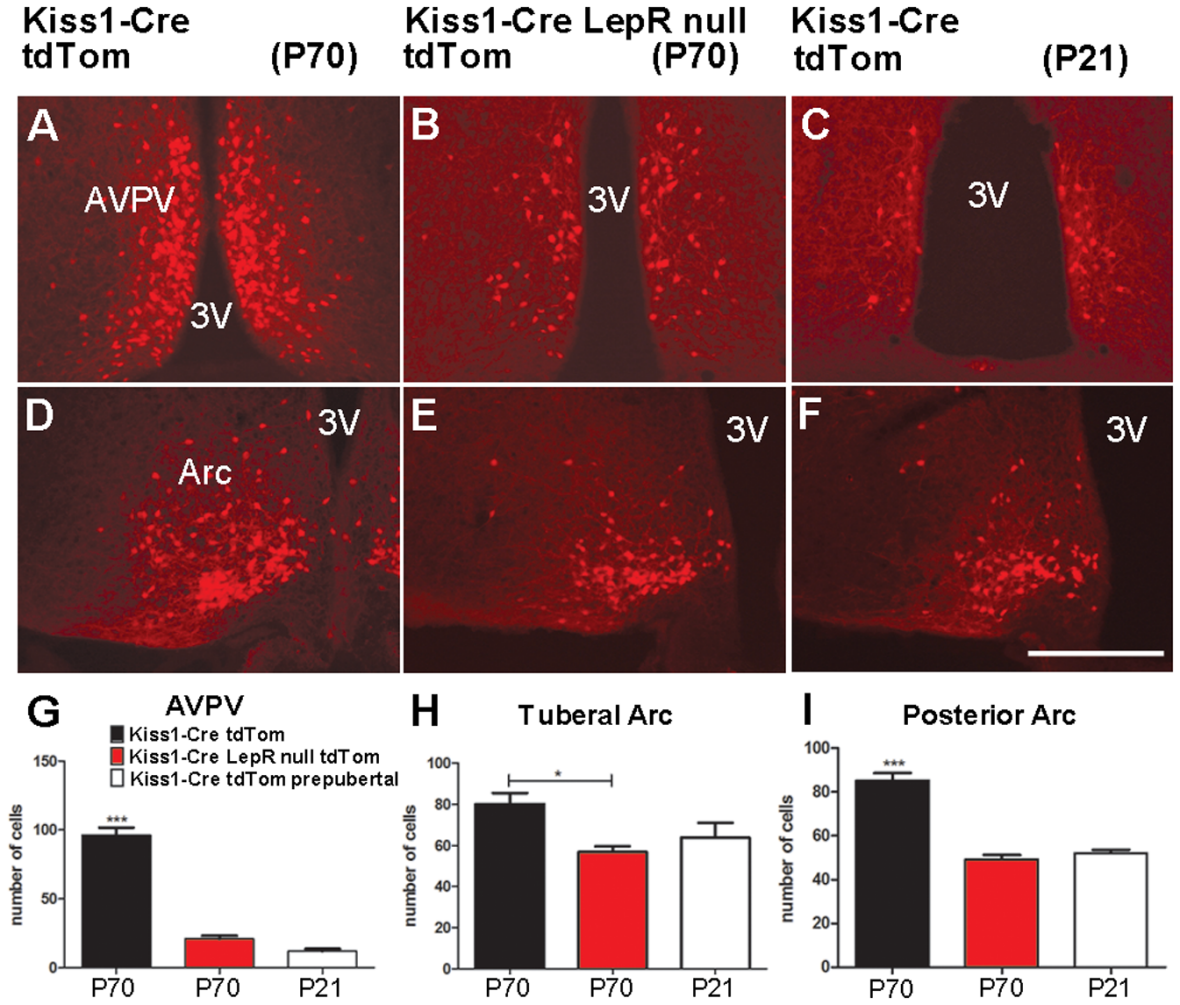
Prepubertal and leptin signaling-deficient mice display decreased numbers of Kiss1 neurons. A-F. Fluorescence photomicrographs showing the distribution of Cre activity (Kiss1 reporter gene) in adult (WT on diestrus and Kiss1-Cre LepR null) and prepubertal (WT) female mice. **G-I.** Bar graphs showing quantification of Kiss1-Cre tdTomato neurons in prepubertal and adult WT female mice and in adult Kiss1-Cre LepR null female mice. Note higher numbers of Kiss1-Cre tdTomato neurons in the anteroventral periventricular nucleus (AVPV) and arcuate nucleus (Arc) of adult WT mice on diestrus compared to adult Kiss1-Cre LepR null and prepubertal WT mice. Scale bar: A–F  =  400 µm. 3V, third ventricle.

**Figure 4:**
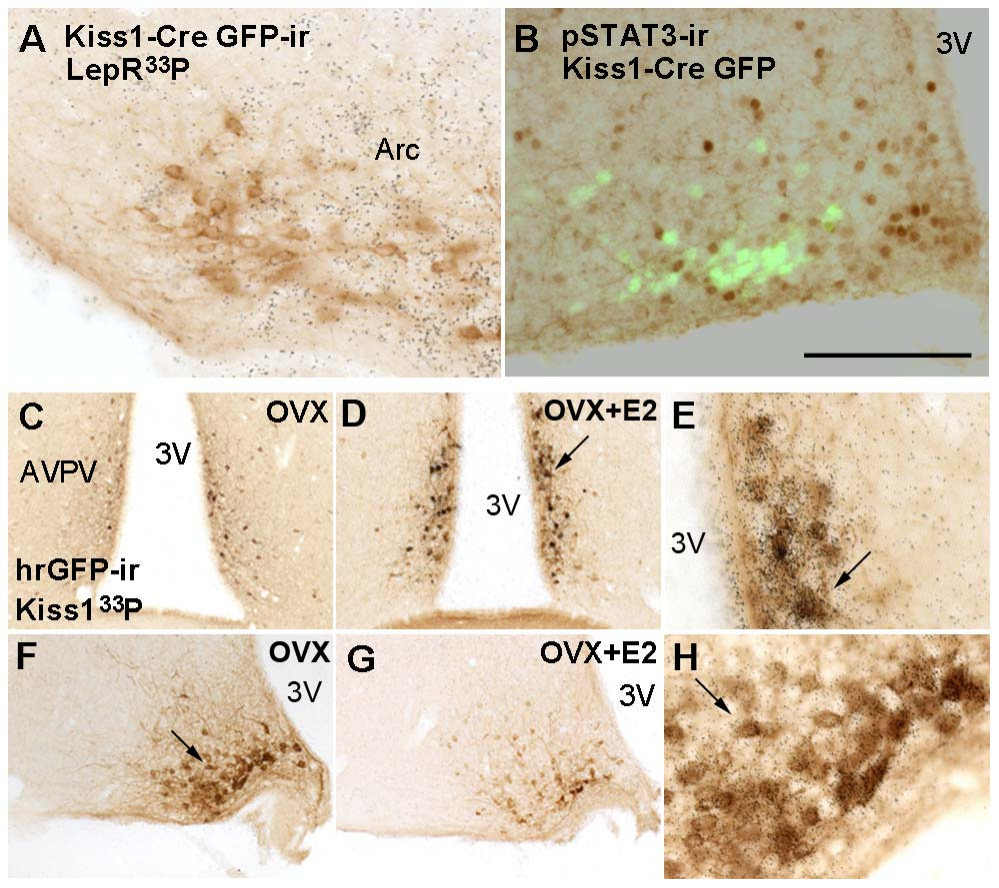
Kiss1 neurons are not responsive to leptin before completion of sexual maturation. A-B. Brightfield and fluorescence photomicrographs showing lack of colocalization of Kiss1-Cre GFP and LepR mRNA (A) or leptin-induced phosphorylation of STAT3 (B) in prepubertal mice. **C-H.** Validation of Kiss1 human renilla GFP (Kiss1-hrGFP) mouse model. To optimize the detection of Kiss1 mRNA, we performed colocalization studies (Kiss1 mRNA and hrGFP) in ovariectomized (OVX) and OVX estrogen primed (OVX+E2) mice. Virtually all Kiss1 neurons in the anteroventral periventricular nucleus, anterior periventricular nucleus (AVPV/PeN) and arcuate nucleus (Arc) of OVX+E2 and OVX mice respectively coexpressed hrGFP immunoreactivity. C-D, F-G. Brightfield photomicrographs showing distribution of hrGFP immunoreactivity in the AVPV and Arc of OVX (C, F) and OVX+E2 (D, G) mice. Note changes in hrGFP expression due to sex steroids manipulation. E, H. Higher magnification of D and F, respectively (arrows indicate same cells), showing coexpression of hrGFP-ir and Kiss1 mRNA in the AVPV of OVX+E2 mice (E) and in the Arc of OVX mice (H). Scale bar: A-B  =  200 µm; C-D, F-G  =  400 µm; E, H  =  80 µm. 3V, third ventricle.

Leptin-deficient *ob/ob* mice display decreased numbers of Kiss1 neurons [10], [36]. Likewise, adult (P70) Kiss1-Cre LepR null female mice showed decreased numbers of neurons expressing Kiss1-Cre activity in the AVPV/PeN and Arc, compared to WT littermates (n =  4–5/group, Fig. 3). Hypothalamic levels of Kiss1 mRNA and numbers of kisspeptin immunoreactive neurons increase during pubertal development [19], [20], [38], [39]. As predicted by their lack of sexual maturation, adult Kiss1-Cre LepR null female mice express similar numbers of Kiss1 neurons as prepubertal mice. Both groups showed decreased numbers of Kiss1 neurons in the AVPV/PeN and Arc compared to adult WT mice (Fig. 3). These observations, added to the lack of re-expression of functional LepR in Kiss1-Cre LepR null mice, raised the hypothesis that coexpression of LepR and Kiss1 mRNAs in hypothalamic neurons arises only after completion of sexual maturation. To test this model, we performed a series of colocalization studies in prepubertal mice using Kiss1-Cre reporter gene and LepR mRNA (in situ hybridization) or leptin-induced pSTAT3, Fos or pSTAT5 immunoreactivities. We found that less than 1% of Kiss1 neurons of prepubertal female mice (1–2 neurons/brain, P21–25, n =  5) coexpressed LepR mRNA and virtually no Kiss1 neurons responded to leptin before puberty (1.6 ± 0.4% pSTAT3-ir+GFP-ir neurons, *P*  =  0.21 compared to saline treated mice; Fig. 4A–B; no dual labeled Fos-ir+GFP-ir or pSTAT5-ir+GFP-ir neurons were identified).

**Figure 5:**
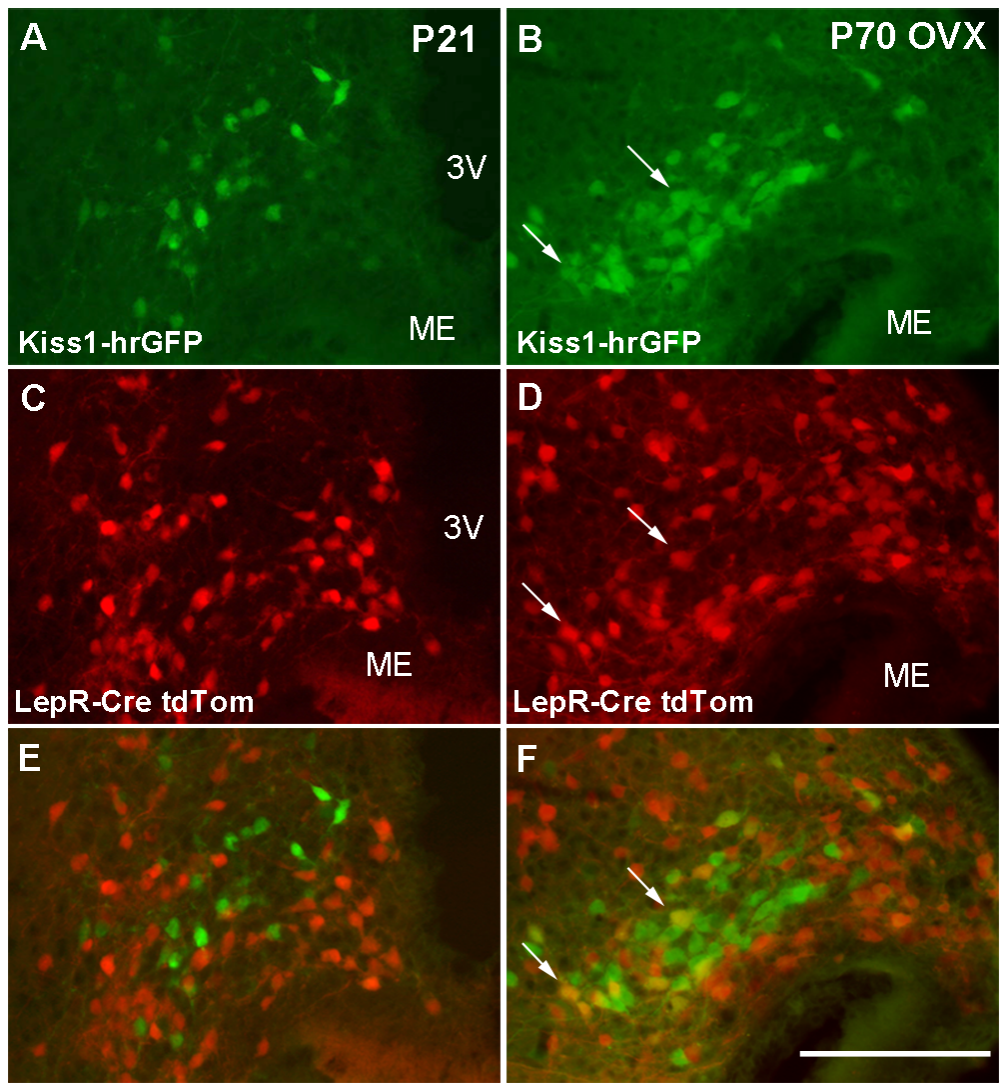
Lack of coexpression of leptin receptor (LepR) and Kiss1 before puberty. A-F. Fluorescence photomicrographs showing the distribution of Kiss1 (hrGFP) and LepR (tdTomato) in prepubertal (21 days of age, P21) (A, C, E) and ovariectomized adult female mice (B, D, F). Note the lack of colocalization of Kiss1 and LepR in prepubertal mice and the higher colocalization rate in ovariectomized adult female mice (arrows indicate dual labeled neurons). Scale bar: A–F  =  200 µm. 3V, third ventricle; ME, median eminence.

In intact mice, Kiss1 and LepR mRNA and protein levels are low and difficult to visualize using standard histological procedures [26], [36], [40]. Thus, to better investigate the temporal coexpression of *Kiss1* and *Lepr* genes, we generated a mouse model in which human renilla green fluorescent protein (hrGFP) is driven by *Kiss1* regulatory elements. The Kiss1-hrGFP mouse model was generated as previously described [25]. Of several potential founders, one line displayed selective hrGFP expression in Kiss1 neurons (Fig. 4C–H). Importantly, hrGFP accompanied the expression of Kiss1 mRNA, i.e., it was also modulated by changes in circulating estrogen levels [10]–[13]. Expression of hrGFP was high in the Arc and low in the AVPV/PeN of ovariectomized mice, and low in the Arc and high in the AVPV/PeN of ovarietomized estrogen-primed mice (Fig. 4C–D, F–G). Kiss1-hrGFP mice were crossed with mice that expressed Cre recombinase from the *Lepr* locus [26] and the R26-tdTomato reporter gene to allow the visualization of Kiss1- and LepR-expressing neurons in a precise and time-controlled fashion. No coexpression of Kiss1 and LepR (hrGFP and tdTomato, respectively) was observed in prepubertal mice (n = 5, Fig. 5A, C, E). As we have previously reported [25], 10–15% (13 ± 1.3) of Arc Kiss1 neurons in ovariectomized female mice coexpressed LepR (Fig. 5 B, D, F) while around 8% (7.8 ± 2.15) of Arc Kiss1 neurons from female mice on diestrus coexpressed LepR. These data reflect the increase in numbers of cells expressing Kiss1 as well as of those expressing LepR in the caudal levels of the Arc in adults compared to prepubertal mice (Kiss1-hrGFP: 18.2 ± 2.4 in prepubertal *vs.* 44.5 ± 4.5 neurons in ovx, *P*  =  0.004; LepR-Cre tdTomato: 54.7 ± 3.8 in prepubertal *vs.* 115.0 ± 8.0 neurons in ovx, *P*  =  0.01). Because cells labeled with Cre-driven reporter genes at these developmental/physiological states represent the accumulation of cells that at some point of the development expressed LepR-Cre, our data indicate that Kiss1 and LepR are coexpressed in a subset of Arc neurons only after pubertal maturation.

Following the description of the relevant role of the kisspeptin-GPR54 system in reproductive physiology and the coexpression of the *Kiss1* and *Lepr* genes in hypothalamic neurons, it has been postulated that the permissive role of leptin in the onset of puberty is relayed by Kiss1 neurons [2], [13], [21]. Here, we demonstrate that leptin signaling in Kiss1 neurons only occurs after completion of sexual maturation, i.e., in adult life. These findings unequivocally demonstrate that kisspeptin neurons do not directly mediate the action of leptin on pubertal development. The physiologic role of leptin signaling in Kiss1 neurons is yet to be demonstrated.

## References

[1] Elias CF (2012) Leptin action in pubertal development: recent advances and unanswered questions. Trends in Endocrinology and Metabolism 23: 9–15. 21978495 10.1016/j.tem.2011.09.002 PMC3251729

[2] Roa J, Garcia-Galiano D, Castellano JM, Gaytan F, Pinilla L, et al (2010) Metabolic control of puberty onset: new players, new mechanisms. Mol Cell Endocrinol 324: 87–94. 20026241 10.1016/j.mce.2009.12.018

[3] Terasawa E, Kurian JR, Keen KL, Shiel NA, Colman RJ, et al (2012) Body Weight Impact on Puberty: Effects of High-Calorie Diet on Puberty Onset in Female Rhesus Monkeys. Endocrinology 153: 1696–1705. 22315448 10.1210/en.2011-1970 PMC3320255

[4] Kennedy GC, Mitra J (1963) Body weight and food intake as initiating factors for puberty in the rat. J Physiol 166: 408–418. 14031944 10.1113/jphysiol.1963.sp007112 PMC1359337

[5] Frisch RE (1985) Fatness, menarche, and female fertility. Perspect Biol Med 28: 611–633. 4034365 10.1353/pbm.1985.0010

[6] Elias CF, Purohit D (2012) Leptin signaling and circuits in puberty and fertility. Cell Mol Life Sci. 10.1007/s00018-012-1095-1 PMC3568469 22851226

[7] Zhang Y, Proenca R, Maffei M, Barone M, Leopold L, et al.. (1994) Positional cloning of the mouse obese gene and its human homologue [published erratum appears in Nature 1995 Mar 30;374(6521: ):479 10.1038/372425a0 7984236

[8] Barash IA, Cheung CC, Weigle DS, Ren H, Kabigting EB, et al (1996) Leptin is a metabolic signal to the reproductive system. Endocrinology 137: 3144–3147. 8770941 10.1210/endo.137.7.8770941

[9] Chehab FF, Lim ME, Lu R (1996) Correction of the sterility defect in homozygous obese female mice by treatment with the human recombinant leptin. Nat Genet 12: 318–320. 8589726 10.1038/ng0396-318

[10] Smith JT, Acohido BV, Clifton DK, Steiner RA (2006) KiSS-1 neurones are direct targets for leptin in the ob/ob mouse. J Neuroendocrinol 18: 298–303. 16503925 10.1111/j.1365-2826.2006.01417.x

[11] Oakley AE, Clifton DK, Steiner RA (2009) Kisspeptin Signaling in the Brain. Endocr Rev 30: 713–743. 19770291 10.1210/er.2009-0005 PMC2761114

[12] Colledge WH (2009) Kisspeptins and GnRH neuronal signalling. Trends Endocrinol Metab 20: 115–121. 19097915 10.1016/j.tem.2008.10.005

[13] Pinilla L, Aguilar E, Dieguez C, Millar RP, Tena-Sempere M (2012) Kisspeptins and reproduction: physiological roles and regulatory mechanisms. Physiol Rev 92: 1235–1316. 22811428 10.1152/physrev.00037.2010

[14] d'Anglemont de Tassigny X, Fagg LA, Dixon JPC, Day K, Leitch HG, et al (2007) Hypogonadotropic hypogonadism in mice lacking a functional Kiss1 gene. Proceedings of the National Academy of Sciences 104: 10714–10719. 10.1073/pnas.0704114104 PMC1965578 17563351

[15] Lapatto R, Pallais JC, Zhang D, Chan Y-M, Mahan A, et al (2007) Kiss1 / Mice Exhibit More Variable Hypogonadism than Gpr54 / Mice. Endocrinology 148: 4927–4936. 17595229 10.1210/en.2007-0078

[16] Topaloglu AK, Tello JA, Kotan LD, Ozbek MN, Yilmaz MB, et al (2012) Inactivating KISS1 Mutation and Hypogonadotropic Hypogonadism. New England Journal of Medicine 366: 629–635. 22335740 10.1056/NEJMoa1111184

[17] de Roux N, Genin E, Carel JC, Matsuda F, Chaussain JL, et al (2003) Hypogonadotropic hypogonadism due to loss of function of the KiSS1-derived peptide receptor GPR54. Proc Natl Acad Sci U S A 100: 10972–10976. 12944565 10.1073/pnas.1834399100 PMC196911

[18] Seminara SB, Messager S, Chatzidaki EE, Thresher RR, Acierno JS Jr, et al (2003) The GPR54 gene as a regulator of puberty. N Engl J Med 349: 1614–1627. 14573733 10.1056/NEJMoa035322

[19] Navarro VM, Fernandez-Fernandez R, Castellano JM, Roa J, Mayen A, et al (2004) Advanced vaginal opening and precocious activation of the reproductive axis by KiSS-1 peptide, the endogenous ligand of GPR54. J Physiol 561: 379–386. 15486019 10.1113/jphysiol.2004.072298 PMC1665361

[20] Shahab M, Mastronardi C, Seminara SB, Crowley WF, Ojeda SR, et al (2005) Increased hypothalamic GPR54 signaling: a potential mechanism for initiation of puberty in primates. Proc Natl Acad Sci U S A 102: 2129–2134. 15684075 10.1073/pnas.0409822102 PMC548549

[21] Blüher S, Mantzoros CS (2007) Leptin in reproduction. Curr Opin Endocrinol Diabetes Obes 14: 458–464. 17982352 10.1097/MED.0b013e3282f1cfdc

[22] Donato J Jr, Cravo RM, Frazao R, Gautron L, Scott MM, et al (2011) Leptin's effect on puberty in mice is relayed by the ventral premammillary nucleus and does not require signaling in Kiss1 neurons. J Clin Invest 121: 355–368. 21183787 10.1172/JCI45106 PMC3007164

[23] Hill JW, Williams KW, Ye C, Luo J, Balthasar N, et al (2008) Acute effects of leptin require PI3K signaling in hypothalamic proopiomelanocortin neurons in mice. J Clin Invest 118: 1796–1805. 18382766 10.1172/JCI32964 PMC2276395

[24] Williams KW, Sohn JW, Donato J Jr, Lee CE, Zhao JJ, et al (2011) The acute effects of leptin require PI3K signaling in the hypothalamic ventral premammillary nucleus. J Neurosci 31: 13147–13156. 21917798 10.1523/JNEUROSCI.2602-11.2011 PMC3319415

[25] Cravo RM, Margatho LO, Osborne-Lawrence S, Donato J Jr, Atkin S, et al (2011) Characterization of Kiss1 neurons using transgenic mouse models. Neuroscience 173: 37–56. 21093546 10.1016/j.neuroscience.2010.11.022 PMC3026459

[26] Scott MM, Lachey JL, Sternson SM, Lee CE, Elias CF, et al (2009) Leptin targets in the mouse brain. J Comp Neurol 514: 518–532. 19350671 10.1002/cne.22025 PMC2710238

[27] Berglund ED, Vianna CR, Donato J Jr, Kim MH, Chuang JC, et al (2012) Direct leptin action on POMC neurons regulates glucose homeostasis and hepatic insulin sensitivity in mice. J Clin Invest 122: 1000–1009. 22326958 10.1172/JCI59816 PMC3287225

[28] Vandenbergh JG (1989) Coordination of social signals and ovarian function during sexual development. J Anim Sci 67: 1841–1847. 2670874 10.2527/jas1989.6771841x

[29] Fu LY, van den Pol AN (2010) Kisspeptin Directly Excites Anorexigenic Proopiomelanocortin Neurons but Inhibits Orexigenic Neuropeptide Y Cells by an Indirect Synaptic Mechanism. J Neurosci 30: 10205–10219. 20668204 10.1523/JNEUROSCI.2098-10.2010 PMC2933146

[30] Bates SH, Stearns WH, Dundon TA, Schubert M, Tso AW, et al (2003) STAT3 signalling is required for leptin regulation of energy balance but not reproduction. Nature 421: 856–859. 12594516 10.1038/nature01388

[31] Zhao AZ, Huan JN, Gupta S, Pal R, Sahu A (2002) A phosphatidylinositol 3-kinase phosphodiesterase 3B-cyclic AMP pathway in hypothalamic action of leptin on feeding. Nature Neuroscience 5: 727–728. 12101402 10.1038/nn885

[32] Niswender KD, Morton GJ, Stearns WH, Rhodes CJ, Myers MG Jr, et al (2001) Intracellular signalling. Key enzyme in leptin-induced anorexia. Nature 413: 794–795. 10.1038/35101657 11677594

[33] Myers MG Jr (2004) Leptin receptor signaling and the regulation of mammalian physiology. Recent Prog Horm Res 59: 287–304. 14749507 10.1210/rp.59.1.287

[34] Qiu J, Fang Y, Bosch MA, Rønnekleiv OK, Kelly MJ (2011) Guinea Pig Kisspeptin Neurons Are Depolarized by Leptin via Activation of TRPC Channels. Endocrinology 152: 1503–1514. 21285322 10.1210/en.2010-1285 PMC3078701

[35] Morris DL, Rui L (2009) Recent advances in understanding leptin signaling and leptin resistance. Am J Physiol Endocrinol Metab 297: E1247–1259. 19724019 10.1152/ajpendo.00274.2009 PMC2793049

[36] Quennell JH, Howell CS, Roa J, Augustine RA, Grattan DR, et al (2011) Leptin Deficiency and Diet-Induced Obesity Reduce Hypothalamic Kisspeptin Expression in Mice. Endocrinology 152: 1541–1550. 21325051 10.1210/en.2010-1100 PMC3206710

[37] Elias CF, Kelly JF, Lee CE, Ahima RS, Drucker DJ, et al (2000) Chemical characterization of leptin-activated neurons in the rat brain. J Comp Neurol 423: 261–281. 10867658

[38] Desroziers E, Mikkelsen JD, Duittoz A, Franceschini I (2012) Kisspeptin-immunoreactivity changes in a sex- and hypothalamic-region-specific manner across rat postnatal development. J Neuroendocrinol 24: 1154–1165. 22458373 10.1111/j.1365-2826.2012.02317.x

[39] Bentsen AH, Ansel L, Simonneaux V, Tena-Sempere M, Juul A, et al (2010) Maturation of kisspeptinergic neurons coincides with puberty onset in male rats. Peptides 31: 275–283. 19944729 10.1016/j.peptides.2009.11.017

[40] Clarkson J, d'Anglemont de Tassigny X, Colledge WH, Caraty A, Herbison AE (2009) Distribution of Kisspeptin Neurones in the Adult Female Mouse Brain. Journal of Neuroendocrinology 21: 673–682. 19515163 10.1111/j.1365-2826.2009.01892.x

